# Myocardial T2* mapping: influence of noise on accuracy and precision

**DOI:** 10.1186/s12968-015-0115-3

**Published:** 2015-02-04

**Authors:** Christopher M Sandino, Peter Kellman, Andrew E Arai, Michael S Hansen, Hui Xue

**Affiliations:** National Heart, Lung, and Blood Institute, National Institutes of Health, DHHS, 10 Center Drive MSC-1061, Bethesda, MD 20892 USA; Department of Electrical Engineering, University of Southern California, Los Angeles, CA USA

**Keywords:** T2* mapping, Iron overload, Mapping, Hemochromatosis, Thalassemia

## Abstract

**Background:**

Pixel-wise, parametric T2* mapping is emerging as a means of automatic measurement of iron content in tissues. It enables quick, intuitive interpretation and provides the potential benefit of spatial context between tissues. However, pixel-wise mapping uses much lower SNR data to estimate T2* when compared to region-based mapping thereby decreasing both its accuracy and precision. In this study, the effects that noise has on the precision and accuracy of pixel-wise T2* mapping were investigated and techniques to mitigate those effects are proposed.

**Methods:**

To study precision across T2* mapping techniques, a pipeline to estimate the pixel-wise standard deviation (SD) of the T2* based on the fit residuals is proposed. For validation, a Monte-Carlo analysis was performed in which T2* phantoms were scanned N = 64 times, the true SD was measured and compared to the estimated SD. To improve accuracy and precision, the automatic truncation method for mitigating noise bias was extended to pixel-wise fitting by using an SNR scaled image reconstruction and truncating low SNR measurements. Finally, the precision and accuracy of non-linear regression with and without automatic truncation, were investigated using Monte-Carlo simulations.

**Results:**

Measured and estimated SD’s were >99.9% correlated for non-linear regression with and without truncation. Non-linear regression with automatic truncation was shown to be the best mapping technique for improving accuracy and precision in low T2* and low SNR measurements.

**Conclusions:**

A method for applying an automatic truncation method to pixel-wise T2* mapping that reduces T2* overestimation due to noise bias was proposed. A formulation for estimating pixel-wise standard deviation (SD) maps for T2* that can serve as a quality map for interpreting images and for comparison of imaging protocols was also proposed and validated.

**Electronic supplementary material:**

The online version of this article (doi:10.1186/s12968-015-0115-3) contains supplementary material, which is available to authorized users.

## Background

T2* mapping is a non-invasive, diagnostic MR tool used to detect iron overload in the brain [1], liver [2], and heart. [3-7] It has been shown that in myocardial tissue, iron concentration is inversely proportional to the transverse relaxation decay rate after electromagnetic excitation also known as T2*. T2* can be measured by sampling points along the T2* decay curve using a gradient-echo sequence and then fitting the points to an exponential function -. This tool allows clinicians to study and diagnose diseases that cause iron overload in tissue such as *β* -thalassemia and hemochromatosis [3-7].

T2* measurement was initially implemented as an ROI-based technique in which an ROI is manually drawn, and a single T2* value is calculated from the average signal intensities at various echo times within that ROI [4,5]. Recently, this idea was extended to pixel-wise mapping which can be produced automatically without user interaction [7]. Parametric maps of T2* values have potential to provide more spatial context than the ROI-based method in that the delineation of adjacent tissues with different T2* values may be less apparent on the raw images. Studies have shown that using pixel-wise T2* maps as opposed to the region-based approach reduces inter- and intra-observer variability [8]. However, pixel-wise T2* mapping suffers from having to use pixel-wise intensity measurements that are much noisier than the averaged intensity measurements used in ROI-based mapping. This noise affects both the precision and accuracy of estimated T2* values.

In this paper, the effects that MR noise has on the precision and accuracy of T2* values estimated using the pixel-wise mapping method are investigated. The automatic truncation method [9] for mitigating noise bias was extended to pixel-wise fitting by using the SNR scaled image reconstruction [10] and truncating low SNR measurements. We hypothesized that automatic truncation would improve T2* measurement accuracy by reducing bias which results from low signal-to-noise ratio at long echo times for cases of low T2*.

The accuracy and precision of the exponential fit is also dependent on the method for fitting. Exponential regression using a non-linear fit is widely used [9,11,12], although some users continue to use the simpler linear regression to the logarithm of the signal, which is computationally faster. We use the non-linear fitting in this work.

A method for evaluating the precision of T2* mapping techniques by estimating the pixel-wise standard deviation (SD) of the T2* based on the fit residuals which were transformed analytically to compute the parametric error is proposed. We hypothesized that pixel-wise SD maps would be a useful quality metric for in-vivo mapping and would also be useful for comparison and optimization of imaging protocols.

## Methods

### Pixel-wise T2* estimation

The T2* recovery curve is described by a 2-parameter mono-exponential of the form:

1 $$ y(TE)=A* exp\left(-\frac{TE}{{T_2}^{*}}\ \right) $$

where y is the measured signal intensity, TE is the echo time, A is the signal amplitude, and T2* is the transverse decay constant.

Signal intensities along the T2* recovery curve are measured using a multi-echo GRE sequence and an exponential regression is performed to fit the measured data to the signal model described above. The regression estimates A and T2* such that the fit residuals are minimized in the least-squares sense. In the case of ROI-based mapping, the regression is performed on one set of average signal intensities inside a manually drawn region for all echo times. In pixel-wise T2* mapping, this process is done on a per-pixel basis generating a parametric map that displays each pixel’s T2* value. This map may be used to evaluate whether the tissues have abnormally low T2* values which is a sign of iron overload.

**Figure 1:**
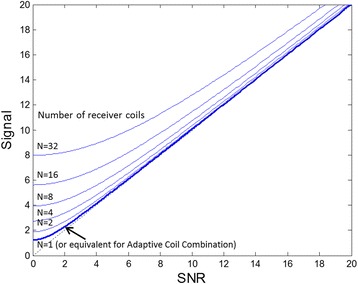
**Plot of SNR vs. Signal intensity for different numbers of coils for root sum of squares combining.** As we increase the number of coil elements, the noise bias (which is the signal at an SNR = 0) increases. Cardiac surface coil arrays can have up to 32-elements, however, the use of adaptive coil combining reduces the effective number of coils to one (see arrow) thereby reducing the noise bias.

Both non-linear and especially linear regression methods tend to overestimate intrinsically shorter T2* values in the range of 0–6 ms due to a noise bias in the acquired data. Noise bias results due to magnitude detection of the normally distributed complex noise, which results in a Rician distribution [13]. For a single coil receiver, the noise bias is approximately 1.25 standard deviations and becomes more significant when root sum-of-squares combining is used with a larger number of coil elements (Figure 1) computed using the non-central chi distribution of magnitude detected multi-echo images (Equation 1–3 of [13]). Using adaptive coil combining [10], the effective number of coils becomes one, which minimizes the noise bias.

**Figure 2:**
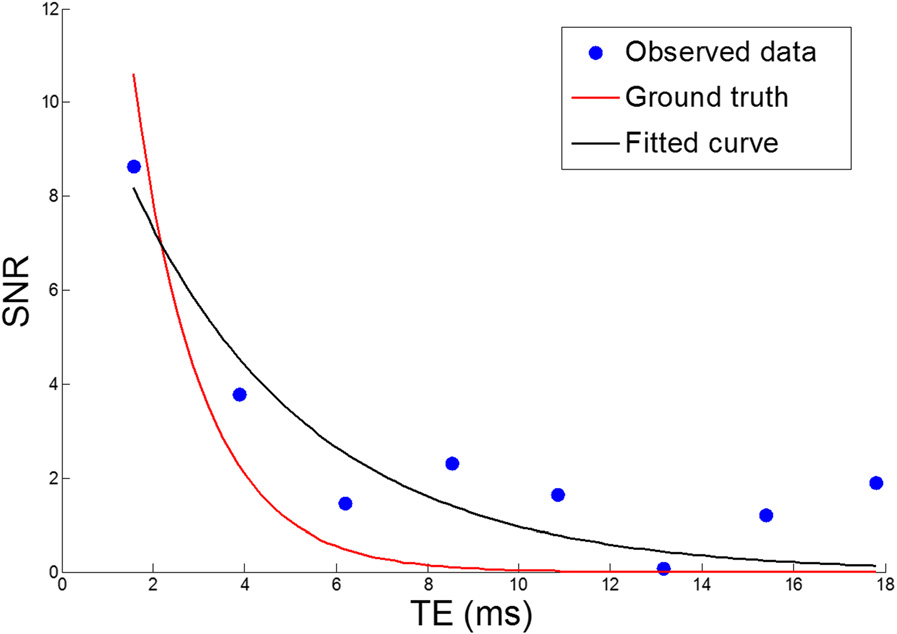
**Observed data for a low SNR measurement** **(SNR =** **15)** with a T2* **of 1.5 ms is simulated.** In the fitted curve, T2* appears artificially longer due to the noise bias which become apparent in the last several points.

Tissues with short T2* have low SNR for long echo times and thus the long echo time measurements are contaminated by the noise bias leading to an apparent non-exponential curve (Figure 2). In this figure, the measured data (in blue) is simulated by adding complex random noise to exponential decay data at the given SNR and T2* value, followed by magnitude detection. For shorter T2* data, if all points are weighted equally, inaccurate and biased points at long echo times will pull the fitted exponential up making the T2* estimate artificially longer. Otto et al. showed that for the log-linear method, the overestimation can be large enough to potentially lead to misdiagnosis [14]. This effect is not observed in intrinsically longer T2* because their curves decay much slower, and low SNR points samples do not occur until later echo times which are not sampled.

To address noise bias, Taigang et al. [9] proposed an automatic truncation method where inaccurate points are truncated based on whether or not that truncation improves the R^2^ metric of the fitted curve. However, this method is only applicable to ROI-based fitting and not pixel-wise due to insensitivity of the R^2^ metric in low SNR measurements. A modified scheme for truncation of inaccurate points that can be applied to pixel-wise fitting is proposed. By utilizing an image reconstruction scheme that outputs images directly in SNR units [10], an SNR threshold is chosen so that all points below it are considered inaccurate and are not used in the regression.

### SD Map theory

The precision of an estimated T2* value can be assessed by estimating the covariance matrix of the fitted parameters. The formulation follows the approach described by Kellman et al. [15,16] for the purpose of T1 mapping. The SD is estimated by computing the covariance matrix for the signal model parameters by inverting a first-order approximation to the parameters’ Hessian matrix:

2 $$ C=\left[\begin{array}{cc}\hfill {\sigma_{{T_2}^{*}}}^2\hfill & \hfill \bullet \hfill \\ {}\hfill \bullet \hfill & \hfill {\sigma_A}^2\hfill \end{array}\right]=inv\left({\displaystyle \sum_{i=0}^{N-1}}\frac{1}{{\sigma_i}^2}\ \left[\begin{array}{cc}\hfill \frac{\partial y\left(T{E}_i\right)}{\partial {T_2}^{*}}\ \frac{\partial y\left(T{E}_i\right)}{\partial {T_2}^{*}}\hfill & \hfill \frac{\partial y\left(T{E}_i\right)}{\partial {T_2}^{*}}\ \frac{\partial y\left(T{E}_i\right)}{\partial A}\hfill \\ {}\hfill \frac{\partial y\left(T{E}_i\right)}{\partial A}\ \frac{\partial y\left(T{E}_i\right)}{\partial {T_2}^{*}}\hfill & \hfill \frac{\partial y\left(T{E}_i\right)}{\partial A}\ \frac{\partial y\left(T{E}_i\right)}{\partial A}\hfill \end{array}\right]\kern0.5em \right) $$

where ${\sigma_{{T_2}^{*}}}^2$ and *σ*_*A*_^2^ are the variances of T2* and A respectively, y (TE_i_) is the signal model equation, and *σ*_*i*_^2^ is the variance of the measurement noise. The partial derivatives of the signal model with respect to its parameters for each echo time TE_i_ were analytically derived and found to be:

3 $$ \frac{\partial y(TE)}{\partial {T_2}^{*}}=A* exp\left(-\frac{TE}{{T_2}^{*}}\right)\ *\left(\frac{TE}{T_2{{}^{*}}^2}\right) $$

4 $$ \frac{\partial y(TE)}{\partial A} = exp\left(-\frac{TE}{{T_2}^{*}}\ \right) $$

The noise is independent and identically distributed across measurements with a normal distribution (*σ*_*i*_ = *σ*) and the noise standard deviation (σ) may be robustly estimated by using the median absolute deviation of the fit residuals [17].

### Imaging

Images were acquired on a 1.5 T clinical MR scanner (MAGNETOM Aera, Siemens AG, Erlangen, Germany) using a multi-echo GRE sequence. A dark blood preparation [18,19] was used to suppress the strong blood pool signal in order to prevent the adjacent blood pool signal from contaminating the desired myocardium signal. For both the in-vivo and phantom studies, a breath-held, segmented acquisition with ECG triggering was used. The multi-echo GRE sequence used gradient flyback for mono-polar readout with 8 echoes (TE = 1.6, 3.9, 6.2, 8.5, 10.8, 13.2, 15.5, and 17.8 ms and TR = 19.7 ms). The matrix size was 256×144 with typical FOV of 360×270 mm^2^ and 8 mm slice thickness. The excitation flip angle was 18°. There were 9 segments acquired each heartbeat. Parallel imaging with factor 2 acceleration was used for a single subject to compare the precision with and without acceleration.

### Processing

A 2-parameter mono-exponential signal model was used to fit all T2* recovery curve data. The downhill simplex minimization proposed by Nelder and Mead [20] was used for the non-linear fit with the initial guess being the solution of the log-linear approach. Images were reconstructed in SNR units and measurements were excluded for all TEs after values fell below a specified SNR threshold. An SNR threshold of 2.5 SDs was used for automatic pixel-wise truncation of measurements. All T2* mapping methods were written in C++ and implemented in the Gadgetron [21] medical image reconstruction framework for inline processing on the scanner.

### Phantom studies

Phantom studies were conducted in order to validate the SD map estimation method. Six agar gel phantoms were created from solutions of 1.5% agarose, cupric sulfate, saline, and varying amounts of Feridex I.V. (Advanced Magnetics Inc., Cambridge, MA), an intravenous contrast agent containing colloids of iron oxide. Depending on the concentration of Feridex, each phantom had a different T2* which was chosen to cover a range of 3 – 30 ms. The phantoms were scanned N = 64 times in order to calculate an estimate of the SD at each pixel and compare it to the mean of estimates provided by the SD maps. ROIs were drawn for each tube and the predicted and calculated SD’s were compared. To perform the fitting, both automatic truncation and no truncation were used to quantify the effect on the standard deviation. We hypothesized that there would be negligible difference between estimated SD’s for both methods.

### In-vivo studies

In-vivo studies were performed with the SNR-scaled reconstruction in order to measure the typical SNR in the septum (N = 20 subjects). SNR estimates were performed using the amplitude fit parameter, i.e., at TE = 0. These studies were run in order to determine the proper range of SNR’s to use in our Monte-Carlo simulations for the noise bias and accuracy studies. Septal ROI values (n = 20) for T2* and SNR were compared with measured SD from the SD maps. This study was approved by the local Institutional Review Board of the National Heart, Lung, and Blood Institute, and all patients gave written informed consent to participate. All patients were referred for CMR assessment of known or suspected heart disease. SD maps were generated for all studies to evaluate their potential for quality maps.

### Noise bias and accuracy experiment

In order to study how precision and accuracy are affected by noise for different regression methods, Monte-Carlo simulations were performed using MATLAB (MathWorks, Natick, MA) in which phantoms of different T2* values and SNR’s were simulated. For each phantom, ground truth decay curves were constructed with T2* = 2, 4, 6, 8, 10, 15, 20, 25 ms and SNR = 15, 25, 35, 45. White noise was added and magnitude detection was used assuming a single coil element corresponding to adaptive combining. Phantoms were then processed with and without automated truncation. For each T2*-SNR combination, N = 32,768 trials were ran and both the bias error and standard deviation of the estimated T2* were calculated to compare methods. Median values were used rather than mean values to avoid contamination by outliers.

## Results

### Phantom validation of SD maps

**Figure 3:**
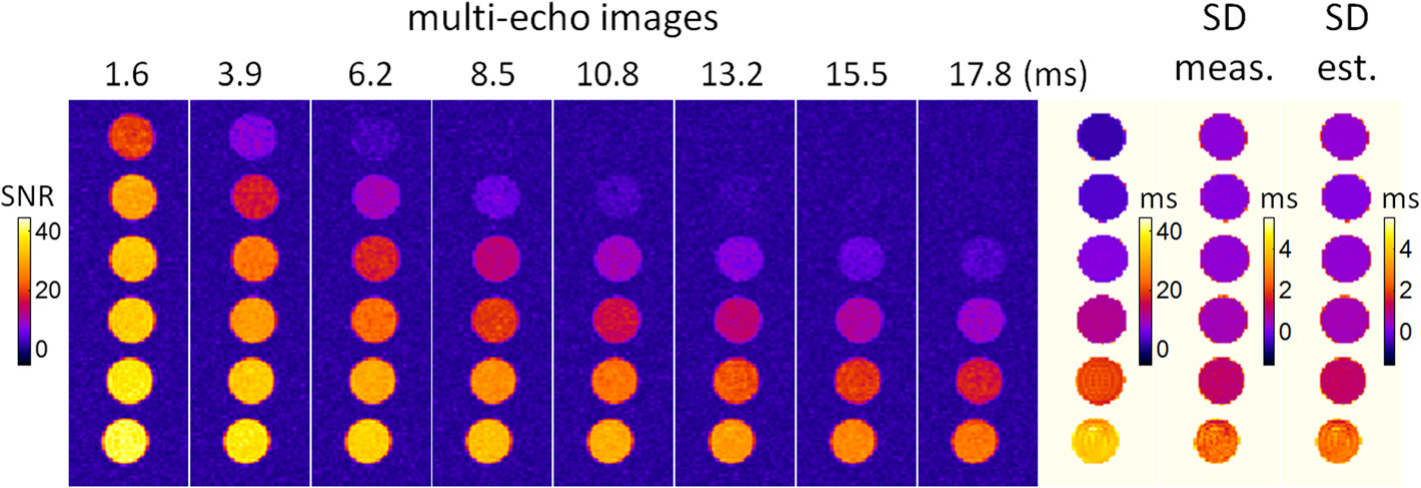
Phantom validation of SD Maps for T2* **using 64 repeated measurements.** Phantoms consisted of 6 tubes with T2* values in 3–30 ms range. Multi-echo images are shown scaled in SNR units for 8 echoes. T2* maps on scale 0–40 ms, and SD maps on scale 0–6 ms.

**Figure 4:**
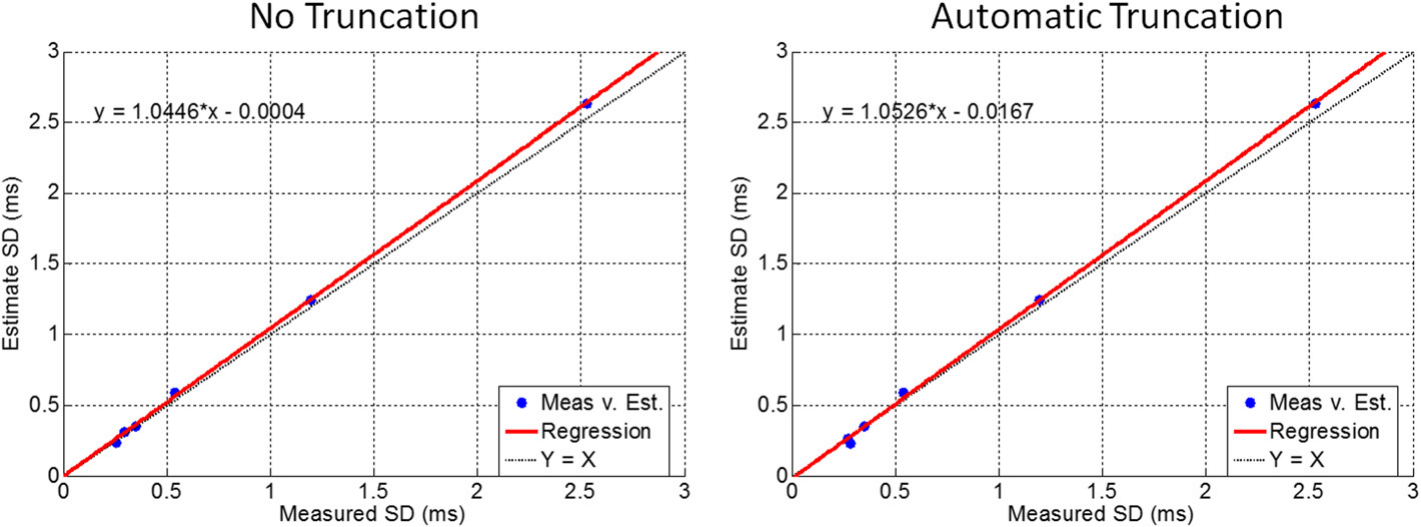
**Measured vs estimated SD’** s for T2* **phantom validation are plotted for exponential fitting method without truncation** **(left)** **for noise bias mitigation and with truncation** **(right).** The estimated SD was in excellent agreement with the measured SD in both cases.

Multi-echo images and corresponding T2* and SD maps are shown in Figure 3 for the phantom study. SNR for the 6 tubes ranged from 40 to 43, calculated at TE = 0 from the exponential fit. Figure 4 plots the estimated SD vs. measured SD for both no truncation and automatic truncation. Measured SD’s and estimated SD’s were >99.9% correlated for both methods. No significant difference was found between the slopes of the automatic truncation and no truncation best fit lines. The measured and estimated SD’s are in close agreement.

### Noise bias and accuracy experiment

**Figure 5:**
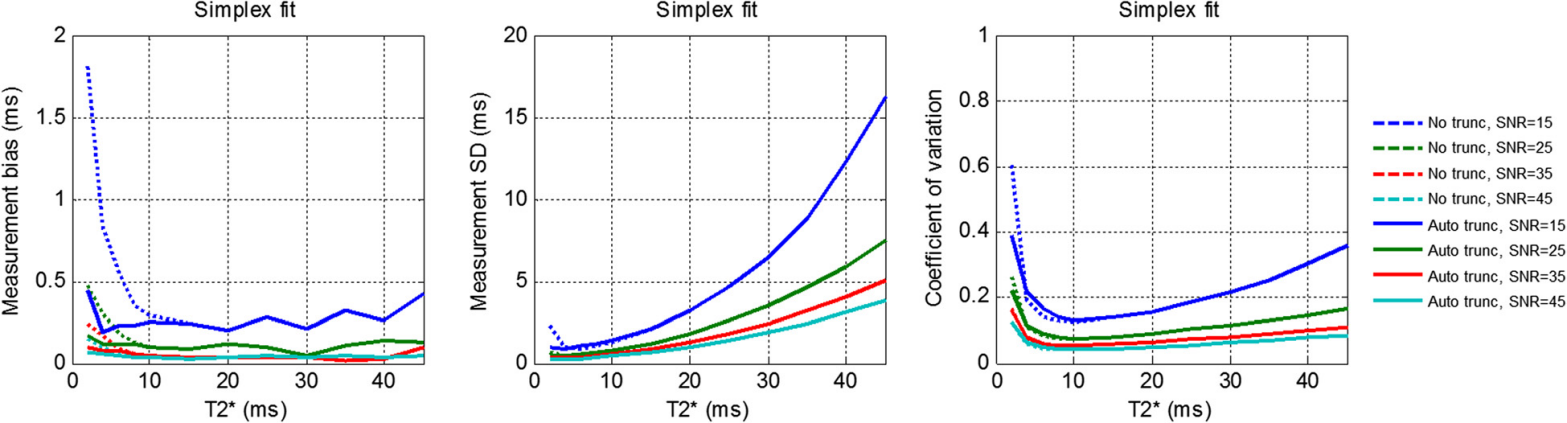
**Monte-** **Carlo simulation of the accuracy** **(bias on left)** **and precision** **(center)** of estimated T2* vs true T2* **and SNR using the simplex fit.** Coefficient of variation (SD/T2*) is shown on right. Dotted lines represent fitting without truncation to mitigate noise bias, and solid lines represent fitting using proposed automatic truncation of values below a specified SNR. Note that bias error increase for low T2* and low SNR. Truncation mitigates noise bias at a very small penalty in precision at low T2*.

Results of Monte-Carlo simulation of the accuracy and precision for T2* fitting are shown in Figure 5. The bias error increases for low T2* and low SNR Truncation mitigates noise bias at a small penalty in precision at low T2*.

### In-vivo measurements

The average SNR in the septal ROI’s was 34.3 ± 7 (N = 20 subjects). The average value of SD measured in the septal ROI was 4.7 ± 1.5 ms (m ± SD, N = 20). In these same ROIs, the average T2* was 37.4 ms. Using the measured value of T2* = 37.4 ms and average SNR = 34.3, the SD predicted by Monte-Carlo calculation was 3.7 ms.

**Figure 6:**
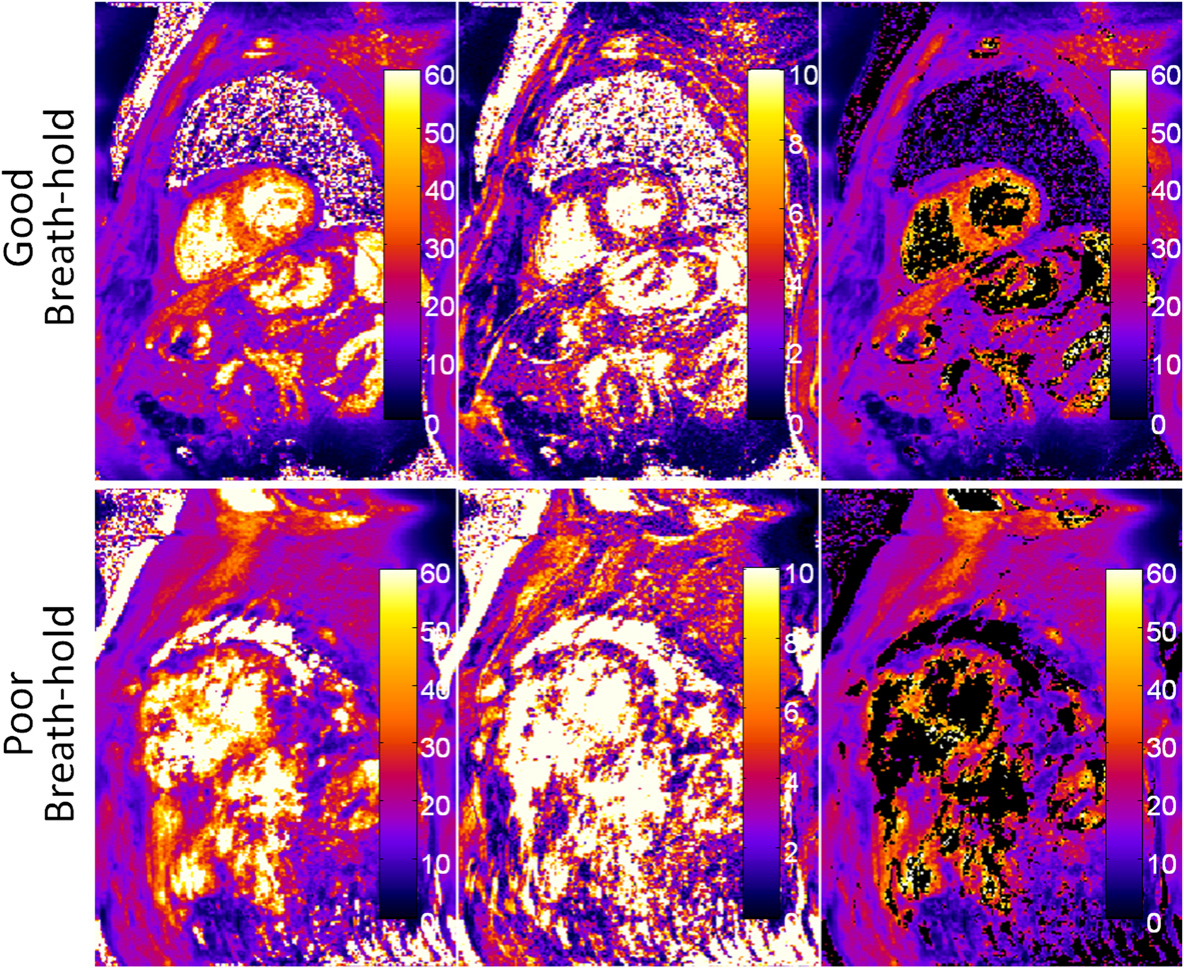
**Examples of in** -vivo short axis view T2* **and SD maps.** The top row shows an example of a good quality breath-hold acquisition while the bottom row shows a poor quality case, which was contaminated by strong motion related ghosting artifacts. Ghosting artifacts in the T2* maps resulted in an increase in the apparent SD (displayed between 0–10 ms). The third column shows the T2* maps in which unreliable points with a high SD were masked. The SD map provides quality control.

Examples of good and poor quality breath-holds for T2* and their corresponding SD maps are shown in Figure 6. The SD in the septal region was approximately 3 ms for the good breath-hold and approximately 30 ms for the poor breath-hold. The SD may be used to mask T2* values that are too noisy (Figure 6, right column) as means of indicating alerting users to low confidence areas.

**Figure 7:**
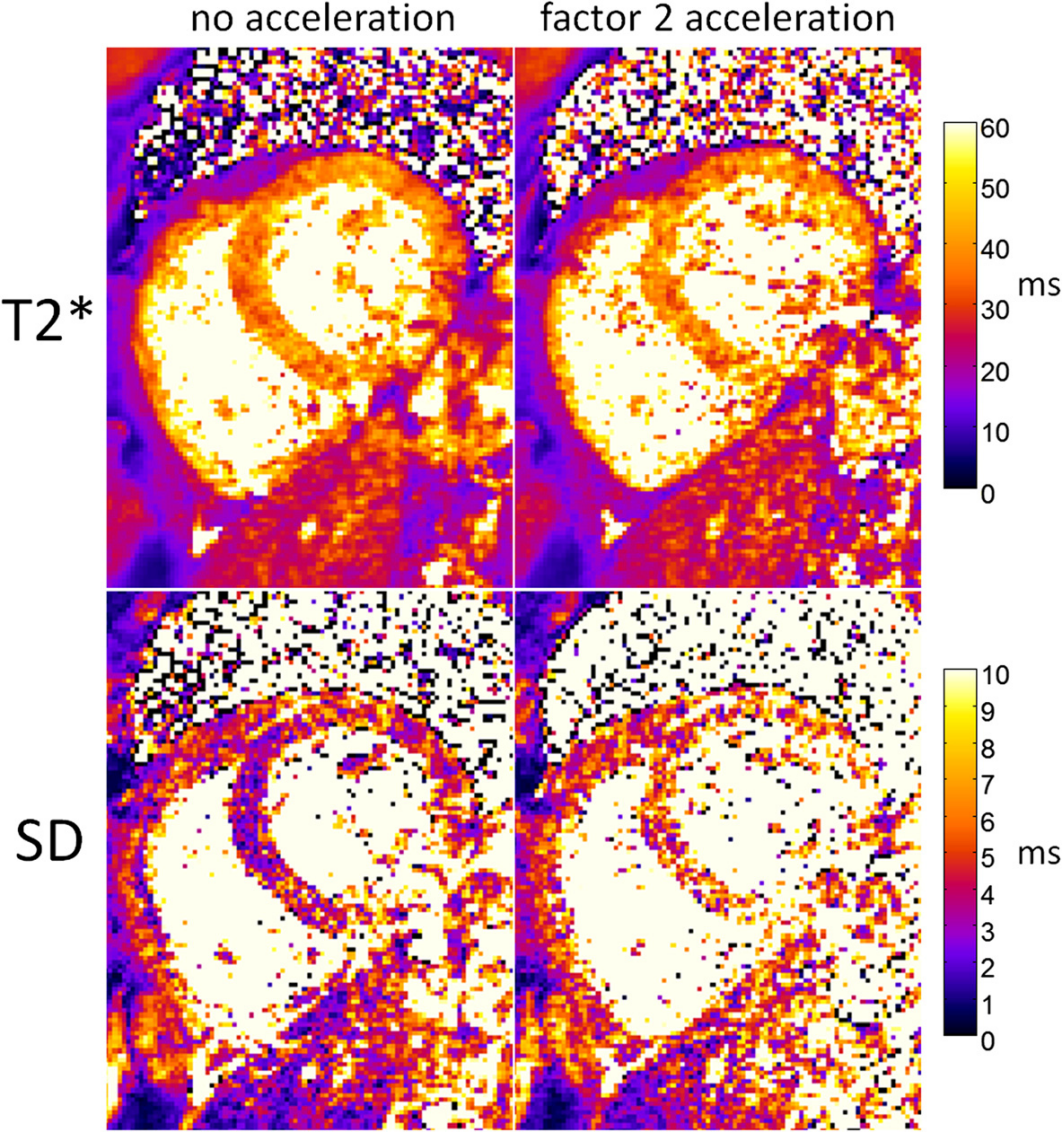
Example of T2* **and SD maps comparing imaging protocols with** **(left)** **and without** **(right)** **parallel imaging acceleration illustrating the trade-** **off in acquisition time and measurement precision**, **and demonstrating how SD maps may be used in comparing performance of imaging protocols.**

T2* and corresponding SD maps (Figure 7) are shown for a single subject comparing 2 protocols: without parallel imaging acceleration (left) and using parallel imaging factor 2 (right). The T2* maps are similar in appearance, but the precision of the accelerated map is degraded as expected by trading off acquisition time for lower SNR. The mean value of T2* in the septum was 37.6 ms without acceleration and 38.93 with acceleration. The pixel-wise SD in the septal region was 3.8 ms without acceleration and 7 ms with acceleration.

## Discussion

### Statement of key findings

We demonstrated that it is possible to estimate pixel-wise SD maps and that they can potentially serve as a quality metric for improving the confidence in interpreting images. SD maps may also be useful for optimizing imaging protocols. As shown in the in-vivo case in Figure 6, poor breath-holding and/or arrhythmias resulted in higher SD estimates due to the ghosting artifacts that cause uncharacteristic variations in the collected recovery curve data. Signal intensities from different pixels are mixed together resulting in recovery curves that are no longer exponential. Note that this non-exponential behaviour is a model mismatch and no longer represents the actual formulation of a noise limited SD. Nevertheless, it is still serves as a useful indication of quality of the T2* map.

It was also demonstrated that the automatic truncation method can be extended to pixel-wise mapping without suffering from the effects of low SNR data. The benefit of truncating low SNR measurements is only relevant for very low T2* and low SNR as seen in the accuracy and precision plots in Figure 5. Longer T2* curves are not truncated at all since points that fall below the noise threshold do not occur until later echo times which are not sampled. It is important to note that truncation may actually cause a small bias (on the order of 0.1 ms) in fitted curves with intermediate T2*’s between 5 and 15 ms depending on the SNR. This occurs because the last sampled points for these curves are at or close to the SNR = 2.5 threshold and so they get truncated. Points at the threshold are just beginning to become biased and may not be completely inaccurate. Truncation of these end points make it seem as if the curve is not decaying as fast causing the T2* to be slightly overestimated. However, this bias is considered slight compared to the gain in precision and accuracy when estimating shorter even T2* (<5 or 6 ms). The T2* bias for the lowest T2* and SNR considered (T2* = 2 ms and SNR = 15) went from 3.6 ms to 0.6 ms after applying truncation of low SNR values.

### SD maps

The measured and estimated SD’s were well correlated and SD values measured in septal ROIs were in close agreement with prediction. The slopes of the best fit lines both with and without truncation were within approximately 5% which is more than sufficient for purpose of a quality metric. This can be attributed to longer T2*s which are difficult to estimate because there are not enough samples to fully characterize the exponential behavior. These curves almost look linear as there are not enough samples at longer echo times where true exponential behaviour is observable.

SD maps serve the purpose of telling us the precision of T2* measurement. If the precision is poor for areas of interest in the image, the clinician would know that they cannot trust it and should re-scan. SD maps could also be useful for discriminating various thresholds for management of diseases, particularly if the T2* values are borderline. SD maps could also potentially be very useful for prototyping and comparing precision between different T2* techniques as in Figure 7.

The SD map only provides information regarding the random error and does not in itself provide any information on systematic bias errors. Although the SD map may indicate obvious artifacts, it will not necessarily reflect bias errors due to mechanisms such as intravoxel dephasing due to susceptibility gradients. Intravoxel dephasing results in a reduction in the apparent T2* which is artifactual, i.e., not a characteristic of the underlying tissue. Signal decay due to intravoxel dephasing is not purely exponential and will result in some model mismatch that may increase the estimated SD. The effect of intravoxel dephasing was not studied and was not considered significant in the septal region, however is important to consider in the lateral wall and near vessels. Intravoxel dephasing is generally more significant at higher field strength, e.g., 3 T but was not considered in this study.

### Effect of truncation on SD

Initially we hypothesized that truncation was only going to affect the accuracy of T2* measurements as it was formulated as a fix for noise bias at short T2*. Calculations of precision and accuracy based on Monte Carlo trials showed that the SD was also lowered when truncation was used for lower T2*. The model mismatch due to noise bias causes regression methods to be highly unstable thereby increasing the SD. Using truncation solves the model mismatch problem, and therefore the SD is slightly lower for very low T2* values. SD is unaffected by truncation for higher T2* values because truncation is only applied to faster decaying curves which have points that fall below the threshold. This reduction in SD corresponds to the improvements in R^2^ as described by Taigang et al. [9].

### Complex vs. magnitude fitting

In the regression methods described in this paper, fitting was performed on the magnitude of complex image data which is the conventional approach. Noise bias may be eliminated by fitting to the complex signal instead of the magnitude, but this requires accurate knowledge of the off-resonance frequency. It is possible to implement complex fitting by estimating the field map from fat-water separated imaging [22-24], but complex fitting was beyond the scope of this paper. Fat water separated imaging combined with T2* estimation offers the additional benefit of a robust elimination of fat from contaminating the water signal.

### ROI measurements

The formulation of pixel-wise SD maps may be extended to ROI measurements provided that the number of independent samples in the ROI is known or can be estimated. The SD of the mean T2* within an ROI is calculated as SD_ROI_ = SD_pixel_/sqrt(N_indep_), where SD_pixel_ is the pixel-wise SD formulated in this work and N_indep_ is the number of independent pixels in the ROI. The number of independent pixels is generally less than the number of image pixels in the ROI due to image interpolation and other effects that blur the spatial resolution. A general framework for calculating the SD for ROI measurements is provided by Hansen, et al. [25], which may be extended to parametric mapping.

### Fitting method

There have been other signal models considered that provide corrections for signal model mismatches such as the offset exponential and the bi-exponential [12], but these require more parameters to be estimated which lowers the regression precision. When fitting to a mono-exponential model, there are two types of regression that are often used: log-linear and non-linear regression. In a log-linear regression, the measured pixel intensities *y* are transformed by log operation, which effectively linearizes the data. The parameters can then be estimated using the ordinary least squares method. Since an analytical solution can be directly obtained by formula, the log-linear method is computationally more efficient than any non-linear method. However, using a log-linear regression is not recommended because its formulation assumes that samples are homoscedastic (uniform variance), which is no longer true for T2* decay curve data after a log transformation. Without homoscedasticity of samples, log-linear regression becomes unstable, which is why non-linear regression was chosen for experiments described in this paper. Non-linear regressions methods employ iterative direct-search minimization algorithms such as Levenberg-Marquardt or downhill simplex to estimate parameters. These methods, however, are computationally slower and require a close initial guess in order to ensure convergence.

### Limitations

One of the limitations of our study was that the imaging protocol was not suitable for very short T2*. The earliest echo time at which recovery curves were sampled was 1.56 ms, which for very short T2*’s (<3 ms) means that there are not enough points sampled before measurements are too noisy and have essentially become the noise bias. In order to sample with shorter echo times and smaller echo spacing it is necessary to reduce the readout resolution. For severe iron overload in the liver where T2* values are much shorter (<3 ms), it is recommended to use a readout matrix of 128 with increased bandwidth.

SNR scaled reconstruction can be useful for determining when to switch protocols to a lower resolution acquisition with shorter initial echo time and echo time spacing. This may be readily implemented by inspection of the number of measurements used in the automatic truncation, which can be output as another image series. If the number of significant measurement reaches 3, then it is an indication of very short T2* and suggests repeating at lower spatial resolution with shorter echo times.

## Conclusions

A method for applying an automatic truncation method to pixel-wise T2* mapping that reduces T2* overestimation due to noise bias was proposed. A formulation for estimating pixel-wise SD maps for T2* that can serve as a quality map for interpreting images and for comparison of imaging protocols was also proposed and validated.

## Abbreviations

GRE: Gradient recalled echo
ROI: Region-of-interest
SD: Standard deviation
SNR: Signal-to-noise ratio
